# Involuntary discharge from drug or alcohol treatment programs in Vancouver, Canada

**DOI:** 10.1186/s12954-024-01036-4

**Published:** 2024-06-21

**Authors:** Kat Gallant, Kanna Hayashi, JinCheol Choi, M-J Milloy, Thomas Kerr

**Affiliations:** 1 British Columbia Centre on Substance Use, British Columbia, Canada; 2 Interdisciplinary Studies Graduate Program, University of British Columbia, British, Columbia Canada; 3 Faculty of Health Sciences, Simon Fraser University, British Columbia, Canada; 4 Department of Medicine, University of British Columbia, British Columbia Centre on Substance Use, 400-1045 Howe Street, Vancouver, BC V6Z 2A9 Canada

**Keywords:** Substance use, Drug treatment, Alcohol treatment, People who use drugs, Treatment discharge, Involuntary discharge

## Abstract

**Background:**

Retention in substance use treatment is essential to treatment success. While programmatic factors are known to influence retention, less is known about the role of involuntary discharges from drug or alcohol treatment programs. Therefore, we sought to identify the prevalence of and factors associated with involuntary discharge due to ongoing substance use.

**Methods:**

Data were derived from two community-recruited prospective cohort studies of people who use drugs in Vancouver, Canada. Generalized estimating equation (GEE) analyses were used to identify variables associated with involuntary discharge from treatment programs due to ongoing substance use.

**Results:**

Between June 2017 and March 2020, 1487 participants who accessed substance use treatment and completed at least one study interview were included in this study. Involuntary discharge from a treatment program due to ongoing substance use was reported by 41 (2.8%) participants throughout the study, with 23 instances reported at baseline and another 18 reported during study follow-up. In a multivariable GEE analysis, involuntary discharge was positively associated with homelessness (Adjusted Odds Ratio [AOR] = 3.22, 95% Confidence Interval [95% CI]: 1.59–6.52), daily injection drug use (AOR = 1.87, 95% CI 1.06–3.32) and recent overdose (AOR = 2.50, 95% CI 1.38–4.53), and negatively associated with age (AOR = 0.93, 95% CI 0.90–0.96). In sub-analyses, participants have most commonly been discharged from in-patient treatment centres (52.2%), recovery houses (28.3%) and detox programs (10.9%), and for using heroin (45.5%) and/or crystal methamphetamine (36.4%).

**Conclusions:**

While involuntary discharge was a relatively rare occurrence, those who were discharged due to active substance use possessed several markers of risk, including high-intensity injection drug use, homelessness, and recent non-fatal overdose. Our findings highlight the need for increased flexibility within treatment programs to account for those who re-initiate or continue to use substances during treatment.

## Background

Canada remains in the midst of an unprecedented public health crisis. In the first nine months of 2022, 5,360 people died of opioid overdose with almost one-third of those occurring in the province of British Columbia [1]. Among the important responses to the ongoing crisis, is the scale up of a range of treatment programs for substance use disorders, particularly those that engage with people who use drugs (PWUD) in treatment. Clinical approaches that deliver evidence-based approaches and reduce social and health-related harms among people who use drugs (PWUD) should do so without judgement or coercion, including those programs that require total and sustained abstinence from substance use [2]. 

Engagement with substance use treatment programs that embody a harm reduction approach has been shown to decrease substance-use related harms including risky behaviours, HIV and hepatitis C virus infections, and overdose deaths [3–5]. Critically, disruptions in treatment can, however, increase the risk of overdose due to reduced tolerance following treatment discharge, as well as increase the risk of experiencing withdrawal symptoms, which can lead to the use of toxic street drugs and increased injection drug use [6–13]. 

Despite the importance of treatment retention, some programs that operate on a strict abstinence-based model have been known to prematurely remove patients from treatment if substance use is detected while enrolled. This phenomenon, referred to hereafter as “involuntary discharge,”^1^ has been identified as undermining treatment outcomes for people with substance use disorders (SUD) [14–16]. Although the potential repercussions of treatment disruption have been documented [6–13, 17], little is known about the prevalence of and factors associated with involuntary discharge in particular. The present study was therefore conducted to identify these gaps and further our knowledge of substance use treatment disruption.

## Methods

### Study design

Data for this analysis were derived from two ongoing prospective community-recruited cohorts of PWUD in Vancouver, Canada: the Vancouver Injection Drug Users Study (VIDUS) and the AIDS Care Cohort to Evaluate exposure to Survival Services (ACCESS) [18, 19]. VIDUS consists of HIV at-risk adults who injected drugs in the month prior to enrollment and ACCESS consists of adults living with HIV who used drugs (other than or including cannabis) in the previous month at enrollment. As previously described, participants are recruited through community-based methods (e.g., self-referral, street outreach, and snowball sampling) in the Downtown Eastside of Vancouver, and complete an interviewer-administered questionnaire at baseline and six-month follow-up visits [18, 19]. The questionnaires are administered in community-based offices located in the Downtown Eastside, which is an area that has a high prevalence of drug use. Given that most participants are residents of this neighbourhood, the findings may not be representative of the broader population of PWUD.

The questionnaires involve the collection of information, on sociodemographic characteristics, patterns of substance use, access and utilization of health services, as well as other relevant social-structural exposures. At each visit, participants also undergo antibody testing (for HIV/HCV infection) or HIV clinical monitoring, as appropriate. Participants receive a CAD$ 40 honorarium at each study visit. The VIDUS and ACCESS studies have received approval from the University of British Columbia/Providence Health Care Research Ethics Board.

### Study sample and measures

The analytical sample for this study was restricted to participants who reported accessing a substance use treatment program in the past six-months and completed at least one study follow-up between June 2017 to March 2020. The primary outcome was self-reported involuntary discharge from drug and alcohol treatment programs due to ongoing substance use in the previous six-months. The main explanatory variable, involuntary discharge, was ascertained from a question: “In the last six months, were you kicked out of any drug or alcohol treatment program because of nicotine, drug or alcohol use?” Socio-demographic characteristics included: Age, self-reported gender (man vs. woman)^2^, race/ethnicity (White vs. Black, Indigenous and People of Colour [BIPOC]), education level (< high school vs. ≥high school diploma), and relationship status (legally married/common law/regular partners vs. others). Substance use-related factors were at least daily use of substance including cocaine, crystal methamphetamine, alcohol, tobacco, cannabis, opioids (including prescription opioids and heroin, and unregulated heroin, fentanyl and down), respectively, daily injection drug use (yes vs. no) and experiencing a recent non-fatal overdose (yes vs. no). Social-structural exposures included recently experiencing homelessness (yes vs. no), recent incarceration (yes vs. no), recently experiencing physical or sexual violence, and having engaged in sex work (yes vs. no) defined as exchanging sex for money, gifts, food, shelter or clothes. Except for the race/ethnicity and education level, all variables were time-updated and referred to the six months prior to each visit.

### Statistical analyses

To determine baseline characteristics, a logistic regression was performed to estimate crude odds ratio, 95% confidence interval, and *p*-value for the continuous variable, age. For the other variables, which are all binary, confidence interval and *p*-value are calculated using normal approximation and Chi-square test, respectively. The test of independence was carried out using the Mann-Whitney U test for age, and Fisher’s exact test for binary variables.

A bivariate generalized estimating equations (GEE) analyses were first conducted to examine potential associations between involuntary discharge and explanatory variables of interest. Variance inflation factors (VIFs) were calculated on predictor variables in the multivariable models to diagnose the multicollinearity. The VIF scores were low suggesting no concern for the existence of multicollinearity in the multivariable model. All statistical analyses were conducted using SAS version 9.4 (SAS Institute, Cary, North Carolina, United States), and all *p*-values were two-sided. In sub-analyses, we used descriptive statistics (n, %) to identify what programs participants were most commonly involuntarily discharged from, and which substances were used that resulted in involuntary discharge.

## Results

Of 1487 eligible participants, 588 (39.5%) were women, 844 (56.8%) were white, 542 (36.5%) were Indigenous, and the interquartile age range was 30 to 53 at baseline. In total, 749 (50.4%) had not completed high school, and 393 (26.4%) were experiencing homelessness. The most common substances used daily included tobacco (83.9%), methamphetamine (80.4%), opioids (28.0%), and cannabis (24.3%). In total, involuntary discharge from a drug or alcohol treatment program due to ongoing substance use was reported by 41 (2.8%) participants at least once during the study period, including 23 instances at baseline and 18 instances during study follow-up. The median number of follow-up questionnaires per participant was 3 (of 6 possible), and the number of observed follow-ups was 4,504. We lost 1,059 participants to follow-up, with a rate of missing follow-up at 0.495.

Participants were involuntarily discharged most commonly for using heroin (45.5%) and/or crystal methamphetamine (36.4%). Treatment programs included in this study were detox and daytox facilities, recovery houses, in-patient and out-patient treatment centres, Narcotics Anonymous/Alcoholics Anonymous/Cocaine Anonymous/SMART, Suboxone programs, counselling, Methadone/Metadol-D/Methadose program, residential community programs, drug treatment court. Notably, participants were most commonly discharged from in-patient treatment centres (52.2%), recovery houses (28.3%) and detox programs (10.9%)^3^, which are all abstinence-based programs that overwhelmingly discharge patients who use unregulated drugs during treatment [20, 21]. While a very small number of local in-patient treatment programs have more relaxed policies in regards to the use of regulated substances (e.g., some programs allow patients to take prescribed opioid agonist treatments and/or smoke tobacco), data were not collected on the specific program(s) each participant accessed meaning we were unable to compare involuntary discharge rates in stricter treatment settings versus others.

**Table 1:** Baseline characteristics and odds ratios of participants among a cohort of PWUD in Vancouver, Canada stratified by having experienced involuntary treatment discharge

Characteristic	Involuntary discharge^a^		*p* – value	
	Yes *n* (%) *N* = 23	No *n* (%) *N* = 1464		
**Median Age (IQR)**	30.36 (30.1–53.1)	42.13 (24.8–33.89)	< 0.001	
**Gender**				
Men	18 (78.3)	874 (59.7)	0.118	
Women	5 (21.7)	583 (39.8)		
**Ethnicity/Ancestry**				
White	17 (73.9)	827 (56.5)	0.161	
BIPOC	6 (26.1)	619 (42.3)		
**Relationship status**				
Legally married/common law/regular partner	4 (17.4)	510 (34.8)	0.126	
Other	19 (82.6)	951 (65.0)		
**Education level**				
High school completion or higher	11 (47.8)	699 (47.6)	0.999	
Less than high school	12 (52.2)	737 (50.3)		
**Employment**				
Yes	4 (17.4)	430 (29.4)	0.305	
No	19 (82.6)	1033 (70.6)		
**Recent homelessness** ^**a**^				
Yes	18 (78.3)	375 (25.6)	< 0.001	
No	5 (21.7)	1076 (73.5)		
**Recent incarceration** ^**a**^				
Yes	4 (17.4)	135 (9.2)	0.330	
No	19 (82.6)	1329 (90.8)		
**Engaging in sex work** ^**a, b**^				
Yes	4 (17.4)	161 (11.0)	0.528	
No	19 (82.6)	1301 (88.9)		
**Daily injection drug use** ^**a**^				
Yes	17 (73.9)	537 (36.7)	< 0.001	
No	6 (26.1)	926 (63.3)		
**Daily unregulated opioid use** ^**a, c,d**^				
Yes	10 (43.5)	407 (27.8)	0.154	
No	13 (56.5)	1056 (72.1)		
**Daily cocaine use** ^**a**^				
Yes	2 (8.7)	49 (3.35)	0.412	
No	21 (91.3)	1415 (96.65)		
**Daily methamphetamine use** ^**a**^				
Yes	9 (39.1)	1186 (81.0)	0.031	
No	14 (60.9)	278 (19.0)		
**Daily alcohol use** ^**a**^				
Yes	2 (8.7)	134 (9.2)	1	
No	21 (91.3)	1329 (90.8)		
**Daily tobacco use** ^**a**^				
Yes	22 (95.65)	1226 (83.7)	0.215	
No	1 (4.35)	235 (16.1)		
**Daily cannabis use** ^**a, d**^				
Yes	7 (30.4)	354 (24.2)	0.579	
No	15 (65.2)	1098 (75.0)		
**Recent overdose** ^**a**^				
Yes	10 (43.5)	271 (18.5)	0.006	
No	13 (56.5)	1192 (81.4)		
**Experiencing physical or sexual violence** ^**a**^				
Yes	7 (30.4)	163 (11.1)	0.011	
No	16 (69.6)	1290 (88.1)		

Abbreviations: BIPOC: Black, Indigenous and People of Colour; CI: confidence interval; IQR: inter-quartile range; PWUD: people who use drugs.

Rounding: percentages may not equate to 100, due to rounding and exclusion of participants responding “not applicable.”

^a^ In the past 6 months.

^b^ includes exchanging sex for gifts, food, shelter or clothes.

^c^ includes the daily use of heroin and/or prescription opioids.

^d^ excludes injected marijuana use.

As indicated in Table 2, in bivariate analyses, factors positively associated with involuntary discharge included identifying as a man (Odds Ratio [OR] = 1.84, 95% Confidence Interval [CI]: 0.92–3.68), experiencing homelessness (OR = 8.61, 95% CI: 4.72–15.72), recent incarceration (OR = 2.82, 95% CI: 1.21–6.58), engaging in sex work (OR = 2.60, 95% CI: 1.26–5.36), daily injection drug use (OR = 2.66, 95% CI: 1.47–4.81), daily methamphetamine use (OR = 1.89, 95% CI: 0.96–3.74), daily tobacco use (OR = 2.80, 95% CI: 0.84–9.39), having recently overdosed (OR = 4.71, 95% CI: 2.69–8.24), and having experienced physical or sexual violence (OR = 2.36, 95% CI: 1.07–5.19). Age (OR = 0.91, 95% CI: 0.89–0.93) was negatively associated with involuntary discharge.

Factors found to be positively associated with involuntary discharge in multivariable analyses included homelessness (Adjusted Odds Ratio [AOR] = 3.22, 95% CI: 1.59–6.52), daily injection drug use (AOR = 1.87, 95% CI 1.06–3.32), and recent overdose (AOR = 2.50, 95% CI 1.38–4.53). Age was negatively associated with involuntary discharge (AOR = 0.93, 95% CI 0.90–0.96).

**Table 2:** Bivariate and multivariable GEE analysis of factors associated with involuntary treatment discharge among a cohort of PWUD in Vancouver, Canada

Characteristic	Unadjusted			Adjusted	
	Odds Ratio (95% CI)	*p* – value		Odds Ratio (95% CI)	*p* – value
**Age**	**0.91 (0.89–0.93)**	**< 0.001**		**0.93 (0.90–0.96)**	**< 0.001**
**Gender**	**1.84 (0.92–3.68)**	**0.085**		1.94 (0.93–4.02)	0.076
(Men vs. Women)					
**Ethnicity**	1.73 (0.87–3.41)	0.117			
(White vs. BIPOC)					
**Relationship status**	0.69 (0.33–1.44)	0.323			
(Legally married/common law/regular partner vs. Other)					
**Education level**	1.26 (0.65–2.42)	0.492			
(High school completion or higher vs. Less than high school)					
**Employment**	1.06 (0.53–2.11)	0.874			
(Yes vs. No)					
**Recent homelessness** ^**a**^	**8.61 (4.72–15.72)**	**< 0.001**		**3.22 (1.59–6.52)**	**0.001**
(Yes vs. No)					
**Recent incarceration** ^**a**^	**2.82 (1.21–6.58)**	**0.017**		0.90 (0.38–2.14)	0.813
(Yes vs. No)					
**Engaging in sex work** ^**a, b**^	**2.60 (1.26–5.36)**	**0.010**			
(Yes vs. No)					
**Daily injection drug use** ^**a**^	**2.66 (1.47–4.81)**	**0.001**		**1.87 (1.06–3.32)**	**0.032**
(Yes vs. No)					
**Daily opioid use** ^**a, c**^	1.70 (0.85–3.42)	0.133			
(Yes vs. No)					
**Daily cocaine use** ^**a**^	2.07 (0.70–6.10)	0.189			
(Yes vs. No)					
**Daily methamphetamine use** ^**a**^	**1.89 (0.96–3.74)**	**0.067**		0.71 (0.36–1.38)	0.314
(Yes vs. No)					
**Daily alcohol use** ^**a**^	1.74 (0.64–4.70)	0.276			
(Yes vs. No)					
**Daily tobacco use** ^**a**^	**2.80 (0.84–9.39)**	**0.095**		2.21 (0.65–7.52)	0.204
(Yes vs. No)					
**Daily cannabis use** ^**a, d**^	1.07 (0.52–2.17)	0.860			
(Yes vs. No)					
**Recent overdose** ^**a**^	**4.71 (2.69–8.24)**	**< 0.001**		**2.50 (1.38–4.53)**	**0.003**
(Yes vs. No)					
**Experiencing violence** ^**a**^	**2.36 (1.07–5.19)**	**0.033**		1.11 (0.51–2.42)	0.795
(Yes vs. No)					

Abbreviations: BIPOC: Black, Indigenous and People of Colour; CI: confidence interval; OR: odds ratio; PWUD: people who use drugs.

^a^ in the past 6 months.

^b^ includes exchanging sex for gifts, food, shelter or clothes.

^c^ includes the daily use of heroin and/or prescription opioids.

^d^ excludes injected marijuana use.

## Discussion

We found that involuntary discharge from substance use treatment due to ongoing substance use does occur, albeit rarely. In our setting, it appears that involuntary discharge primarily impacts younger people, those experiencing homelessness, people who inject drugs every day, and those who have experienced a recent overdose. While the number of events suggests that involuntary discharge is a relatively infrequent occurrence, it is worth exploring and addressing given its potential negative impacts. For instance, treatment disruption and sudden withdrawal are linked to an increased risk of overdose due to reduced tolerance, which places high-intensity drug users at greater risk due to their pre-treatment high tolerance and likelihood of re-engaging in previous levels of substance use upon discharge [6–11, 22]. Although temporal relationships are difficult to discern in this observational study, it is concerning that non-fatal overdose was associated with involuntary discharge, as those experiencing a non-fatal overdose are known to be at heightened risk for fatal overdose [23]. 

Despite periods of re-initiation and remission generally being considered a normal part of the ongoing process of recovery, abstinence-based programs remain a prominent option among substance use treatments [14, 24]. Proponents of involuntary discharge often believe that it promotes a value system which maintains drug-free program integrity [14]. Programs with this ideology create an environment where patients who re-initiate are considered to have broken the code of conduct. In such settings, program rule violators cannot be allowed to remain in the program while others have adhered to the rules, otherwise, it is perceived as implying approval for drug use during treatment [14]. Programs that create and perpetuate such environments, place high moral value on sobriety and place reward systems on remaining drug-free (e.g. earning sobriety “chips” or “tokens” in 12-Step programs), which can discourage program retention if individuals re-initiate.

The potential for involuntary discharge within abstinence-based programs presents an additional challenge for those experiencing homelessness, an exposure linked to higher likelihoods of involuntary discharge in our multivariable model. Given the multiple daily stressors and risks homeless individuals experience, including poverty, poorer physical and mental health, high-intensity substance use, and higher risk of assault, injury and death, the abrupt move to abstinence is likely challenging and, as mentioned, potentially dangerous to their health [25–30]. 

Opponents of involuntary discharge from treatment programs recognize re-initiation as a normal part of chronic conditions, including substance use disorders. As Woody and colleagues (2007) argue, health systems do not typically stop providing care to patients with other chronic conditions, such as diabetes, hypertension and other mental health conditions and, as such, prioritizing retention in treatment is essential, particularly for reducing mortality [31]. Similar arguments have been made, regarding involuntary discharge as a form of “clinical abandonment” and violating the “first do no harm” rule [16]. In this framing, clinical abandonment adheres to the Code of Ethics (2021) written by the NAADAC, the Association for Addiction Professionals, which states that: [32]

Addiction professionals shall not abandon any client in treatment. Providers who anticipate termination or interruption of services to clients shall notify each client promptly and seek transfer, referral, or continuation of services in relation to each client’s needs and preferences.

Comparing involuntary discharge with clinical abandonment emphasizes the importance of treatment retention and the continuum of care, which may not happen if patients are discharged abruptly and involuntarily. It also positions the preferences and goals of PWUD as a critical component of treatment retention and improved outcomes. Programs that consider these preferences typically allow PWUD more autonomy in their care through non-judgemental and non-coercive strategies that ‘meet people where they are at,’ – a core tenant of harm reduction [33]. 

An example of this integrated treatment and harm reduction approach is the Community Oriented Substance Use Programme (COSUP) in Tshwane, South Africa [34, 35]. The COSUP is South Africa’s first publicly funded community-based harm reduction program for people who use drugs. Established in 2016, the program has established 17 service sites across Tshwane, promoting itself as an “evidence-based, public-health informed, feasible alternative to an abstinence-based approach to substance use.” [34] The program offers traditional services often provided by recovery-oriented services such as counselling and shelter, but it also offers linkages to care, opioid substitution therapy services, needle and syringe services, as well as social services and skills development. To note, the program does have involuntary treatment discharge policies, however, the continued use of substances does not warrant an involuntary treatment discharge.

Perinatal substance use services across North America have also begun to embrace a harm reduction approach to treatment. The Perinatal Addiction Treatment Clinic in Hawaii, United States (“PATH clinic”) provides prenatal and postpartum care in an out-patient clinic, as well as mental health services, parenting and birthing classes, and social services, and has shown improved health and wellness among participants [36]. While the clinic values abstinence and meeting attendance through contingency management, there is no punishment or removal from the program for continued drug use.

Given the need to support individuals to remain in substance use treatment programs, we recommend the following responses programs can take to reduce the risks of involuntary discharge. First, education and training to staff regarding respecting individual care plans, the varied definitions of recovery, and evidence-based approaches and strategies to treat substance use disorders. Second, provide multiple treatment options and services for both the patient and the patient’s family. Third, develop and continuously modify individualized treatment plans to accommodate changes in patient goals and their current substance use status. Fourth, connect patients to community-based services during and after their time in the program. In particular, connections made during treatment help to build trust between patients and the service providers and increases the likelihood that individuals will access these services upon discharge [37, 38]. Finally, if continued substance use or relapse is ultimately against program policy and must involve an involuntary discharge, the continuum of care should be maintained by implementing strategies to facilitate connections to other community-based supports and services upon discharge.

Like any study, there are limitations to this analysis. Most of the data shown were obtained via self-report and therefore may be susceptible to response and other biases. Additionally, it is possible that some influencing variables are not accounted for, such as personal motivation to reduce substance use when entering treatment.

To create a dichotomized gender variable for the analysis, we condensed gender identity categories into two variables: “women” and “men”, in part due to the low representation of non-binary individuals in our study. However, we recognize the homogenization of various gender identities into singular categories may obscure the unique impacts that transgender, Two-Spirit and other persons who identify outside of the gender binary may face in regards to involuntary treatment discharge. We recommend future research allow for greater gender diversity in their analyses of this topic. Finally, this study provides a descriptive analysis that identifies the prevalence of and factors associated with involuntary discharge. However, it does not address and cannot comment on the consequences of involuntary discharge for these participants. Given the potential for harm arising from involuntary treatment discharge, it would be useful for future research to investigate these impacts with people with lived experience of this phenomenon. Additionally, we recommend further investigation into treatment settings where these discharges are occurring at higher rates. In-depth interviews with treatment providers, participants, as well as an examination of program policies related to involuntary discharges could provide a more holistic understanding of this phenomenon.

## Conclusions

In summary, while involuntary discharges were rarely reported by our study participants, those who were discharged due to active substance use possessed several markers of risk, including high-intensity injection drug use, homelessness, and a history of recent non-fatal overdose. Our findings highlight the need for increased flexibility within drug and alcohol treatment programs to account for those who re-initiate or continue to use substances during treatment.

## Data Availability

The data that support the findings of this study are not publicly available due to the potential to identify participants and break confidentiality, but may be available from the corresponding author on reasonable request.

## Footnotes

1 Referred to elsewhere as “administrative discharge” or “premature discharge.”

2 The gender variable is further categorized in cohort study surveys. For this analysis, we dichotomized the variable by combining “male” and “transgender male” into the category “men,” and “female,” transgender female,” “Two-Spirit,” and “others” – all others who identify outside of the gender binary – into the category “women.”

3 Substance use treatment program descriptions: In-patient treatment centres are defined as “Time-limited, live-in intensive treatment (typically 60–90 days) for individuals experiencing substance use-related challenges. Treatment includes group and one-on-one counselling, medical consultations, as well as life skills training, family support programs”; recovery houses are defined as “a stable, short-term place to live for individuals who are participating in a substance use recovery program”; and detox programs are defined as “withdrawal management, also known as detox, is a short-term service (up to 7 days) that provides clinical support to people withdrawing from substances. Withdrawal management takes place in different settings, including community, hospital, or home (with clinical team support).”
